# MCPH1: A Novel Case Report and a Review of the Literature

**DOI:** 10.3390/genes13040634

**Published:** 2022-04-02

**Authors:** Stefano Giuseppe Caraffi, Marzia Pollazzon, Muhammad Farooq, Ambrin Fatima, Lars Allan Larsen, Roberta Zuntini, Manuela Napoli, Livia Garavelli

**Affiliations:** 1 Medical Genetics Unit, Azienda USL-IRCCS di Reggio Emilia, 42123 Reggio Emilia, Italy; marzia.pollazzon@ausl.re.it (M.P.); roberta.zuntini@ausl.re.it (R.Z.); livia.garavelli@ausl.re.it (L.G.); 2 Department of Bioinformatics, Institute of Biochemistry, Biotechnology and Bioinformatics (IBBB), The Islamia University of Bahawalpur, Bahawalpur 63100, Pakistan; muhammad.farooq@iub.edu.pk; 3 Department of Biotechnology, Institute of Biochemistry, Biotechnology and Bioinformatics (IBBB), The Islamia University of Bahawalpur, Bahawalpur 63100, Pakistan; 4 Department of Cellular and Molecular Medicine, University of Copenhagen, Blegdamsvej 3B, 2200 Copenhagen, Denmark; ambrin.fatima@aku.edu (A.F.); larsal@sund.ku.dk (L.A.L.); 5 Department of Biological and Biomedical Sciences, Aga Khan University, Karachi 74800, Pakistan; 6 Neuroradiology Unit, Azienda USL-IRCCS di Reggio Emilia, 42123 Reggio Emilia, Italy; manuela.napoli@ausl.re.it

**Keywords:** microcephaly, MCPH1, MRI, SNP array, simplified gyral pattern

## Abstract

Microcephaly primary hereditary (MCPH) is a congenital disease characterized by nonsyndromic reduction in brain size due to impaired neurogenesis, often associated with a variable degree of intellectual disability (ID). The genetic etiology of MCPH is heterogeneous and comprises more than 20 loci, nearly all following a recessive inheritance pattern. The first causative gene identified, *MCPH1* or *Microcephalin*, encodes a centrosomal protein that modulates chromosome condensation and cell cycle progression. It is also involved in DNA damage response and telomere maintenance in the nucleus. Despite numerous studies on *MCPH1* function, MCPH1-affected individuals are rare and the available clinical reports are not sufficient to define the natural history of the disease. Here, we present a novel patient with congenital microcephaly, ID, language delay, short stature, and other minor features such as strabismus. magnetic resonance imaging revealed ventriculomegaly, simplified gyral pattern in the frontal lobes, and a neuronal migration defect. Genetic testing detected a homozygous deletion of exons 1–8 of *MCPH1*. We compare the patients’ characteristics with a list of features from MCPH1 cases described in the literature, in an effort to provide additional clues for a comprehensive definition of disease presentation and evolution.

## 1. Introduction

Primary microcephaly (PM) is a congenital neurodevelopmental disorder characterized by a small brain and a head size at least 3 standard deviations (SD) below the mean of age, sex, and ethnicity-matched controls, as defined by the measurement of occipital-frontal circumference (OFC) [1]. PM can have different genetic etiologies and manifests either as a feature in a variety of syndromes or as an isolated condition. The subgroup of nonsyndromic PM, termed “microcephaly primary hereditary” (MCPH), also shows considerable genetic heterogeneity, with at least 28 associated or likely associated loci (see [2], OMIM: *MCPH1-28*, and [1] for a recent review). For the most part these loci follow an autosomal recessive inheritance model, but rare autosomal dominant forms are emerging (OMIM #617520, #619179, #619180) and some complex digenic or triallelic cases have been suspected [3]. Prevalence is estimated to be one in 100,000 live births, but it may be as high as 1:10,000 in populations where consanguineous marriages are a common practice [4].

MCPH genes are implicated in various steps of mitotic regulation and are particularly enriched in pathways related to centrosome biogenesis or spindle assembly and function [1,4]. Defects in these genes share the common pathogenetic mechanism of impairing neurogenesis, resulting in lateral and radial reduction in the neocortex [5,6]. Since the neocortex is the site of higher brain functions, MCPH associates with a variable degree of intellectual disability (ID), usually in the absence of other gross neurological anomalies.

Variation in the *Microcephalin 1* gene (*MCPH1*; OMIM *607117) accounts for 1–9% of MCPH cases, depending on the population considered [7,8,9]. *MCPH1* covers a genomic region of 241 kb on chromosome 8 (8p23.1) and comprises 14 coding exons. It is transcribed into two major isoforms, similarly expressed in various human tissues: a full-length form and a form lacking exons 9–14 (deltae9−14) [10]. *MCPH1* mRNA shows high expression in the fetal brain and particularly in the lateral ventricular zone, where the neural progenitors that develop into the neocortex originate [9]. The full-length isoform encodes an 835 amino acid protein containing three BRCA1 C-terminal (BRCT) domains, one at its N terminus and two in tandem at its C terminus. BRCT motifs are protein–protein interaction domains that play an essential role in BRCA1 and other proteins involved in cell cycle control, DNA damage repair, and genome stability [11].

MCPH1 is highly pleiotropic. It is capable of nuclear localization and has been recognized as a proximal factor in the hierarchy of DNA damage response pathways. It mediates the recruitment of the repair machinery into nuclear foci at DNA damage sites [12,13] and is involved in telomere maintenance [14]. MCPH1 also localizes in the centrosome and coordinates its maturation with cell cycle progression through the CHEK1-CDK1 pathway [15,16]. It modulates the loading of condensin II on chromosomes through interactions mediated by its N-terminal and central domains, and it promotes efficient chromosome alignment to the spindle during prometaphase [17,18]. *MCPH1* defects lead to the uncoupling of chromosome condensation from centrosome maturation and cell cycle progression, resulting in spindle misalignment and mitotic delay [19]. Upon cytogenetic analysis, a portion of the mitotic cells from individuals bearing *MCPH1* defects shows a distinctive prophase phenotype defined as premature chromosome condensation (PCC) [20].

Efficient neurogenesis requires a rapid proliferation phase of the neural precursors. The mitotic delay and the irregular spindle orientation caused by defects in *MCPH1* lead to an early switch of cell division from symmetric (self-renewal) to asymmetric (neuronal specification), resulting in a depletion of the progenitor pool and in microencephaly [6,21]. Since neural progenitors have a high proliferative index and rely heavily on DNA repair to correct replication errors, impairment of the DNA damage response properties of MCPH1 have also been considered in the pathogenesis of microcephaly [22].

Here, we describe a novel patient with MCPH showing a homozygous deletion in *MCPH1* and compare it with a review of the *MCPH1*-associated PM cases reported in the literature to date. This detailed information can offer further insights on the evolution of the disease.

## 2. Results

### 2.1. Clinical Report

The patient, a 10-year-old boy, is the only child of a healthy consanguineous couple (first cousins) originating from Gujrat, in northern Pakistan. The mother and father, 37 and 42 years old at the time of delivery, had elected for medical termination of a previous pregnancy due to unspecified cerebral malformations of the fetus. During the second pregnancy, prenatal ultrasound revealed microcephaly and intrauterine growth retardation, and the mother was treated with several drugs prescribed in Pakistan (calcium, folic acid, secnidazole, chorionic gonadotropin, ranitidine, progesterone, aspirin, prednisolone). Growth arrest at the 34th week of pregnancy required her hospitalization; fetal magnetic resonance imaging (MRI) was performed to investigate the microcephaly, but the report was not available to us.

The patient was born by caesarean section at the 38th week of gestation and showed the following parameters: weight 2005 g (<3rd centile, −2.5 SD), length 43 cm (<3rd centile, −2.7 SD), OFC 28 cm (<<3rd centile, −3.5 SD), Apgar score 9 at both 1 and 5 min. Soon after birth he showed hypertonia, failure to thrive, and transient hypoglycemia, for which he was admitted to the Neonatal Department and fitted with a feeding tube.

**Figure 1:**
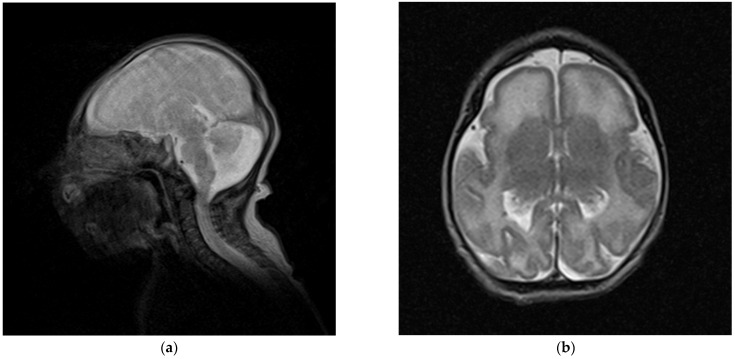
Brain MRI (turbo spin echo T2) performed at 3 days of life. (**a**) Sagittal view: microencephaly with flat frontal bone and enlarged posterior fossa with large cisterna magna. (**b**) Axial view: posteriorly enlarged lateral ventricles and fronto-polar lissencephaly with increased cortical thickness.

**Figure 2:**
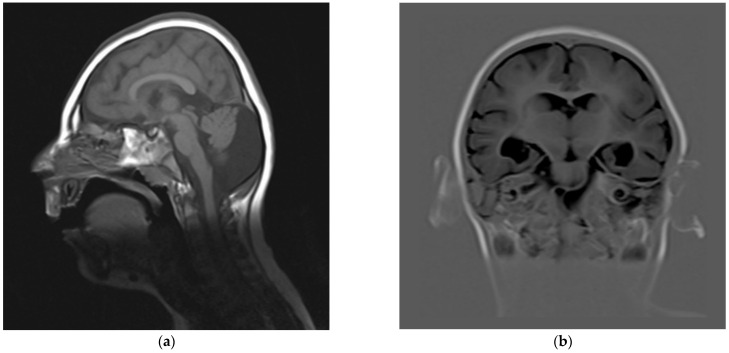

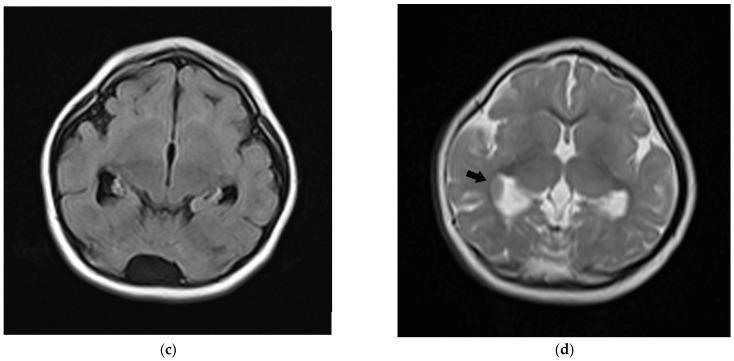
Brain MRI follow-up at 2 years 1 month of age. (**a**) Spin echo T1, sagittal view: enlarged posterior fossa with large cisterna magna. (**b**) Inversion recovery, coronal view: posteriorly enlarged lateral ventricles. (**c**) Fluid-attenuated inversion recovery, axial view: enlarged ventricles and fronto-polar simplified gyral pattern. (**d**) Turbo spin echo T2, axial view: right periventricular nodular heterotopia (arrow).

Repeated brain MRI at 3 days of life (Figure 1) and at 2 years and 1 month (Figure 2) confirmed the microencephaly and showed a bilateral simplified gyral pattern in the fronto-polar region, enlarged sylvian cisterns, dilated temporal horns of the lateral ventricles, and enlarged posterior fossa with a large cisterna magna causing an imprint on the cerebellar vermis and hemispheres. The second MRI also revealed periventricular nodular heterotopia (PNH), the largest nodules being evident in the right paratrigonal region (Figure 2d).

Echocardiography at 1 month showed restrictive ventricular septal defect and thickened aortic and pulmonary valve cusps. Serial electroencephalography (until 2 years and 1 month revealed moderate organization and unusual activity, without clear paroxystic anomalies. Ophthalmological evaluation showed exotropia (right > left) but was otherwise normal. Abdominal ultrasound and audiometric evaluation were normal.

**Figure 3:**
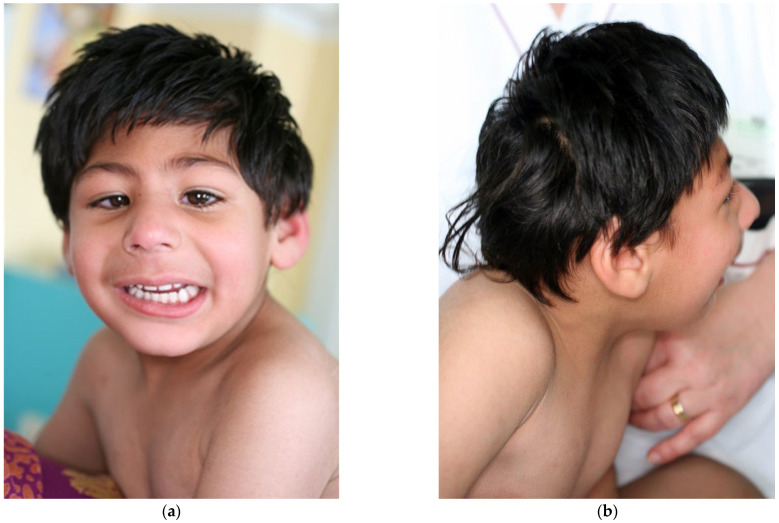
Facial features of our patient at 4 years 7 months of age: (**a**) front, (**b**) profile.

The patient was first referred to our Medical Genetics Unit at the age of 2 years and 8 months. A report from the Pediatric Neurology and Psychiatry service indicated a mild delay in achieving motor milestones: the patient attained trunk control at 7 months and was able to walk alone at 18 months. Morphologically (Figure 3), he presented with a low and sloping forehead, highly arched eyebrows, narrow palpebral fissures, epicanthus, exotropia, and large ears with left preauricular pit. Hands and feet were normal.

Throughout his follow-up, both microcephaly and a non-progressive growth delay were noticeable. At the last physical examination at 10 years of age, his parameters were: weight 35 kg (50th–75th centile), height 124 cm (<3rd centile, −2.3 SD), OFC 46.5 cm (<<3rd centile, −5 SD), and body mass index 22.8 kg/m^2^. The OFCs of his parents were 57.5 cm for the mother and 58 cm for the father, both >97th centile (+2–3 SD).

Neuropsychiatric evaluation reported profound ID. Language is absent: the patient is only capable of a few mispronounced words in the Urdu language. He has partial sphincter control. During preschool he has had isolated episodes of aggressive behavior. At present he exhibits a sociable disposition, although he still displays some hyperactivity.

### 2.2. Molecular Diagnosis

Karyotype on peripheral blood was normal male (46,XY) and comparative genomic hybridization (CGH) array at 100 kb resolution was normal. Because of the simplified gyral pattern, fluorescence in situ hybridization (FISH) analysis of the 17p13.3 locus containing the *LIS1* gene and Sanger sequencing of the *DCX* gene were performed, but results were normal.

Given the suspicion of a PM, blood samples from the patient and both parents were sent to a specialized laboratory for further genetic testing. Here, homozygosity mapping was performed using sequence-tagged site (STS) markers for the genomic loci mainly associated with microcephaly in the Pakistani population at the time: *MCPH1* (gene *MCPH1* on chromosome 8p23.1), *MCPH2* (*WDR62* on 19q13.12), *MCPH3* (*CDK5RAP2* on 9q33.2), *MCPH9* (*CEP152* on 15q21.1, previously classified as *MCPH4*), *MCPH5* (*ASPM* on 1q31.3), and *MCPH6* (*CENPJ* on 13q12.12). Our proband only showed homozygosity at the *MCPH1* locus (Figure S1a). Sanger sequencing of the entire *MCPH1* coding sequence was attempted, but PCR amplification of exons 1–8 failed consistently even after repeated experiments, suggesting a large deletion not detectable by standard CGH array (Figure S1b).

Fine mapping of the region with high resolution single nucleotide polymorphism (SNP) array genotyping analysis (Genome-Wide Human SNP Array 6.0, Affymetrix) revealed a 61 kb homozygous deletion comprising the first 8 exons of *MCPH1*: (GRCh37) chr8:6246755_6307795del (Figure S1c). Confirmation and familial segregation of this copy number variant was performed via long-range PCR using primers spaced about 73 kb apart (forward: 5′-CTCTGAAACGCCCCAGTATG-3′, ~27 kb upstream of exon 1; reverse: 5′-GACATCGGTAAACCCAAAGC-3′, within intron 8). Gel electrophoresis of the amplicons alongside a 1 kb DNA ladder showed an 8–9 kb product in the proband and in both parents, while no amplification occurred in control samples (Figure S1d). Long-range PCR analysis along with the STS markers data suggested that the parents were both heterozygous for the deletion. This was later confirmed independently by multiplex ligation-dependent probe amplification (MLPA) analysis on DNA from saliva samples (SALSA MLPA kit P355, MRC-Holland; see Figure S2).

Signed informed consent was obtained from the subject’s parents for genetic testing and for the publication of pictures and clinical data.

## 3. Discussion

The role of *MCPH1* in the pathogenesis of MCPH has been recognized since the early 2000s and its functions have been studied extensively, but knowledge about the natural history and evolution of the disease is still limited.

In the literature, reports of affected individuals specifically associated with *MCPH1* defects are rare. We compiled the available reports in a single list (Table 1 and Table S1) for a total of 31 independent families. These cases include our proband and a report in which *MCPH1* may act as a contributing cause of microcephaly through interaction with a biallelic *TRAPPC9* variant [3]. A study in which a heterozygous missense variant was observed in affected twins without further familial segregation or functional analysis was excluded [23]. Additional cases reported in ClinVar or other databases were also excluded, since the clinical description was absent or insufficient.

The patient described here is quite illustrative for the MCPH1 condition. His microcephaly is progressive, with an OFC below −3 SD at birth. The only notable neurodevelopmental sign is ID with an absence of structured language—a direct consequence of the reduced extent of the cerebral cortex, particularly pronounced in the frontal lobe.

Although microcephaly at birth is the pathognomonic sign of MCPH1, in the literature neonatal evaluation was only available for 12 families; 9 of which had proper OFC measurements that ranged between −1.5 and −5 SD. Postnatal OFC, in individuals aged a few months to more than 40 years, ranged from −3 to −12 SD. In a large study on MCPH in the Iranian population [7], intrafamilial variability was noted to be within 2 SD. When considering MCPH1 alone (Table 1), variability spanned no more than 1 SD in families with OFCs of −5 SD or less; but it reached 4−5 SD in a few pedigrees with more severe microcephaly. Half of the families included affected individuals with OFC below −6 SD, and cases with microcephaly below −8 SD were reported in about one third of the pedigrees. These comparisons are indicative, since the exact OFC at age was often unavailable. Age at measurement was sometimes given as a range for the whole pedigree and sometimes omitted, especially in the earliest studies

Neurological alterations of the brain in addition to its reduced size were detected. The simplified gyral pattern observed in some cases is likely a direct consequence of impaired cortical development, as a function of cell proliferation and mechanical compression dynamics [42]. Various reviews over the years have assumed this to be a recurrent MCPH-associated feature also including MCPH1, but in fact early examples referred to *ASPM*-related MCPH5 patients exclusively [5,16], and only one *MCPH1*-related case was observed before 2012 [24]. A mild gene-specific contribution to cortical folding has been suggested for *ASPM* [43], while no correlation data is available for *MCPH1*. Out of 11 *MCPH1*-related cases with a reported diagnostic imaging of the brain, three did not show any significant clinical finding, while five (including our proband) had anomalies in sulcation/gyrification. A defective cell proliferation phase during neurogenesis is expected to associate with a reduced cortical surface area, possibly leading to simplified gyral pattern, while mantle thickness should appear normal [5], but this has been explicitly confirmed in only two cases. In most reports, neuroradiological images were unavailable and it was unclear whether the observed cortical reduction or atrophy also involves a variation in thickness. A systematic review of these cortical anomalies is hindered by the fact that they are dependent on age, as the brain continues its postnatal development. As an example, repeated MRI in our patient attests to a neuroradiological evolution: at birth, fronto-polar lissencephaly with increased cortical thickness was reported, while at two years of age the anomaly of the frontal lobes was described as a simplified gyral pattern (Figure 1b and Figure 2c,d). The frontal polarity agrees with the reviews for *ASPM* [5] and with two siblings described in an early report of *MCPH1* [20,24]. Interestingly, these siblings and our case also presented with PNH in the ventricular area, indicating a neuronal migration defect. In agreement with the timeline of brain development, PNH was only evident in our proband’s second MRI (Figure 2d).

Infratentorial anomalies such as cerebellar hypoplasia, with or without an enlarged posterior fossa, have been described, especially in *ASPM*-, *CDK5RAP2*-, and *WDR62*-related microcephaly [21]. Hypoplasia of the cerebellum was observed in three MCPH1 families (Table 1), while our proband had a distinct enlargement of the cisterna magna leaving an impression on the cerebellum, mistaken for hypoplasia in the imaging performed at birth. Impaired cortical development and sulcation anomalies may lead to an enlargement of the ventricular system, as noted in the lateral ventricles and sylvian cisterns of three MCPH1 reports, including our own.

ID appeared highly variable in MCPH1 patients, accounting for many factors—sociocultural, genetic, and even subjective evaluation. There was no clear correlation with head size and/or brain anomalies. About 25% of the patients had severe to profound ID, mostly in the group with OFC < −5 SD, but some individuals were described as mildly impaired despite severe microcephaly. Poor or absent language usually led to an evaluation of severe ID or worse, but it was also reported in one family with mild ID. Besides speech, a slight delay in developmental milestones was noted in eight families, with no clear connection to the severity of ID. In our patient an IQ structure test could not be administered, and evaluation was based on the Lapmer scale.

MCPH1, similar to other MCPH subtypes, may present with additional features of variable type and severity. These could be related in part to the emerging of recessive traits, since nearly all pedigrees show some degree of consanguinity. Variability is well exemplified by the comparison of our patient with the case described by Hemmat et al. [39]: although both have a similar deletion involving the first 8 exons of *MCPH1* and no other annotated genomic element, Hemmat’s patient presented with milder microcephaly and no structural brain anomalies. Strikingly, in a pair of teenager siblings OFC ranged between −5 in the sister, who had normal intelligence, and −12 SD in the brother, with severe ID [36]. The authors reasonably suggested that sex may be a discriminant factor, and that the variant type—a splicing defect leading to a small in-frame deletion—may be particularly prone to individual variability. Unfortunately, determining whether birth sex correlates with the severity of microcephaly in MCPH1 can be problematic, because most studies only offer a generic clinical description at the pedigree level rather than at the individual level.

Growth delay was observed in affected individuals related to most of the MCPH loci, although quite rarely and with variable frequency. Out of the 31 MCPH1 families reported in Table 1, stature was reliably evaluated in 19 and described as normal in 8 (counting two siblings at the lower end of the normal range). The remaining families included patients with stature −2 to −3 SD below average (even −5 SD in one case): a variable reduction in height was a relatively frequent feature, observed in about one-third of the pedigrees, and did not correlate with OFC. In these families, MCPH1 bordered on microcephalic primordial dwarfism (MPD) syndromes. Interestingly, the genes responsible for MPDs are often involved in pathways closely related to the MCPH genes: *PCNT*, encoding a centrosomal protein interacting with MCPH1, is associated with Meier–Gorlin syndrome, while most Seckel syndrome patients display variants in *ATR*, encoding a modulator of DNA damage response acting downstream of MCPH1. Some genes can actually be responsible for either nonsyndromic PM or Seckel syndrome, such as *CENPJ* and *CEP152*, required for centriole biogenesis and centrosomal function [1]. Growth delay is consistent with the intrinsic pathogenic mechanism of MCPH. Neurogenesis demands rapid proliferation of the neural progenitor cells and is therefore particularly sensitive to mitotic delay [6,22]. Occasionally, an alteration in the spatiotemporal requirement of mitotic factors, possibly based on multifactorial cues rather than specific genotype–phenotype correlations, may lead to an overall delay in body growth, as suggested by experimental mouse models [44].

**Figure 4:**
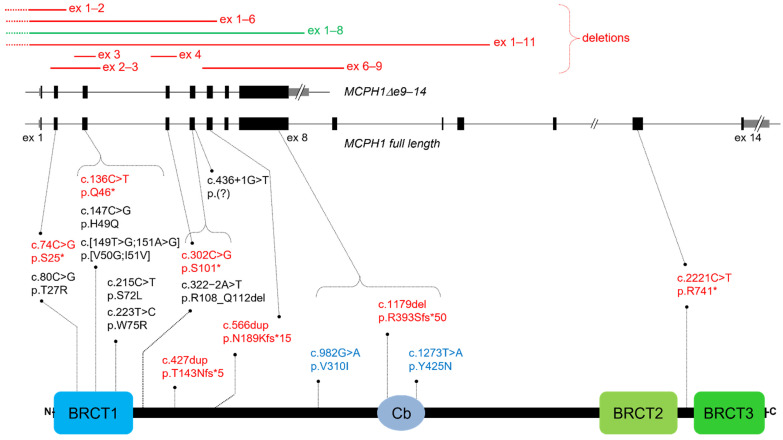
Schematic representation of the two main *MCPH1* transcripts and of the full-length protein, on which variants reported in Table S1 have been mapped. ex: exon; gray boxes: UTRs; black boxes: exons; *: stop codon; del: deletion; dup: duplication; BRCT: BRCA1 C-terminal domain; Cb: condensin II binding region; text in red: frameshift/nonsense variants; text in blue: compound heterozygous variants; text in green: deletion identified in our patient.

Pathogenic variants in MCPH1 patients can be missense, nonsense, frameshift, splicing, and small or large deletions (Figure 4) [6]. Generally, they result in a loss of function, with no clear genotype–phenotype correlation. However, some variants may be hypomorphic and preserve part of the protein function, as appears to be the case for NM_024596.5:c.80C > G, p.T27R, associated with mild cellular and clinical phenotypes [26]. Amino acid substitutions are concentrated within or near the essential BRCT1 domain, with a few exceptions, such as a compound heterozygous variant affecting the central condensin II binding region [40].

The N-terminal BRCT1 domain and the central portion of MCPH1 are considered responsible for centrosome function and proper coupling of chromosome condensation with the cell cycle: both the constitutive deltae9–14 isoform and BRCT2/3-deleted constructs, but not BRCT1-deleted constructs, can rescue the PCC phenotype, at least in part [10,17,45]. On the other hand, the C-terminal BRCT2/3 domain, specific to the full-length isoform, is primarily associated with DNA damage response [5,6,11]. A transgenic mouse model expressing an MCPH1 protein lacking BRCT3 displayed cells with PCC phenotype, but brain size and body weight were unaffected [46]. An *MCPH1* nonsense variant escaping RNA decay and leading to a truncated protein that lacks BRCT3 was described in two siblings with PM [3]. Of note, the same biallelic variant was also observed in their healthy sister. It was proposed that the *MCPH1* defect acted as a modifier of a homozygous variant in *TRAPPC9*, a gene usually associated with postnatal microcephaly, which was heterozygous in the unaffected sister. Taken together, these data suggest that the BRCT2/3 domain, though still important for protein function, is not a limiting factor during neurogenesis, and variants in this region may have a hypomorphic effect which does not result in microcephaly. Incidentally, the deletion in our patient eliminates the exons corresponding to the MCPH1deltae9−14 isoform. Although in this case both transcripts are suppressed, it would be interesting to find out whether there is a genotype–phenotype correlation in patients with the reverse alteration, i.e., molecular defects only affecting the full-length transcript.

Given the role of MCPH1 in cell cycle regulation and DNA repair, its possible role as a tumor suppressor has been considered extensively. MCPH1 acts upstream of ataxia telangiectasia mutated (ATM), a major mediator of DNA damage response [47,48]. Germline variants in *ATM* are responsible for the cancer predisposition syndrome Ataxia Telangiectasia [49]. However, families harboring *MCPH1* pathogenic variants do not seem to manifest increased cancer susceptibility. On one hand, some *Mcph1* knockout mouse models have shown genomic instability and increased incidence of tumors [50], while knockdown experiments on human cell lines via RNA interference indicated impaired formation of irradiation-dependent DNA repair foci [12]. On the other hand, patient-derived cell lines with hypomorphic and null *MCPH1* variants show just a modest reduction in foci formation, possibly as a result of redundant cellular pathways [51]. It has been hypothesized that in MCPH1 patients the G2/M checkpoint in response to DNA damage is still competent, but causes a longer delay in cell cycle re-entry, which might explain the microcephaly phenotype in the absence of a clear cancer predisposition.

## 4. Conclusions

Even though MCPH1 has been recognized as a nosological entity for more than 20 years, it is still difficult to describe its natural history or establish genotype–phenotype correlations. Reports of repetitive OFC measurements from the same individual over time are rare, and in fact OFC at birth is unavailable in several cases. Finding accurate birth data, exhaustive neuropsychiatric evaluations, or repeated IQ tests can be problematic. It is often impossible to define the exact prevalence of features, since several studies describe pedigrees as a whole or through the example of an index case. This applies to occasional findings, but also to OFC and ID.

The present case report highlights the difficulty in collecting consecutive data from the clinical history of even a single individual and attempts to piece together the available information from previous publications in order to offer a comprehensive overview specific for *MCPH1*-associated patients. In conclusion, the most relevant aspects for patient management are limited to microcephaly, short stature, and neurodevelopmental-related features such as ID, language impairment, and behavioral problems. Our patient showed a favorable evolution of behavior over time. Neuroradiological findings are not uncommon in MCPH1, but in order to consider specific suggestions for follow-up, a longitudinal study of brain MRIs by an expert panel would be appropriate.

This report also reiterates the value of either high resolution SNP array or next-generation sequencing for the molecular diagnosis of MCPH. While microcephaly panels would be appropriate, whole exome/genome sequencing should be preferred, given the considerable genetic heterogeneity of this condition with many loci still undiscovered.

## Figures and Tables

**Table 1:** Summary of the clinical findings in families with *MCPH1*-related primary microcephaly described in the literature.

Study Ref.	Patients #^a^	Variant Types	Birth OFC, in SD	OFC (at Age), in SD	Stature, in SD	DD/ID	MRI Findings	Other Findings	Ethnic Origin
[8,9,24]	7 (2 peds)	ns	MC	−5 to −10 (13–28 y)	variable	mild to moderate ID	reduced cortex size, mild cerebellar hypoplasia	no	Pakistani (Mirpur)
[20,24]	2 sibs	fs	−3.5 to −4	−8 to −10 (5−7 y)	−2 to −5	DD, profound ID (poor or no language)	reduced cortex size, pachygyria, mild cerebellar hypoplasia, PNH, ventriculomegaly	slightly upslanted palpebral fissures, thin upper lip (hyperreflexia in one sib)	nk
[25]	7 (2 peds)	linkage only	nk	−7 to −10 (0−50 y entire MCPH cohort)	nk	mild to moderate ID (language delay?)	nk	sloping forehead in some cases	Pakistani (north)
[26]	1	mis in BRCT1	−2	−3 (6 y)	−0.5 (normal)	mild ID (normal language, mild fine motor delay)	nk	mild PCC effect; 2–3 toe syndactyly, upslanted palpebral fissures	German and nk
[7,27]	7 (1 ped)	del ex 1−6	nk	−3 (18−32 y)	−2.5 to −3	moderate ID	nk	no	Iranian (north)
[28,29]	1	ns	nk	−9 (16 y)	−3	moderate ID	nk	craniosynostosis, ptosis, mild micrognathia, mild exotropia	Danish
[7]	2 related	del ex 4	nk	−10 to −11	nk	moderate ID	nk	no	Iranian
[7]	3 related	fs	nk	−6	nk	moderate ID	nk	no	Iranian
[7]	3 related	del ex 2−3	nk	−6 to −8	nk	mild to moderate ID	nk	no	Iranian
[7]	2 related	splice site	nk	−9	nk	severe ID	nk	no	Iranian
[7]	4 related	mis in BRCT1	nk	−7 to −9	nk	moderate ID	nk	no	Iranian
[7]	2 related	del ex 3	nk	−6 to −10	nk	mild to moderate ID	nk	no	Iranian
[7]	3 related	mis in BRCT1	nk	−6 to −7	nk	mild to moderate ID	nk	(ataxia and autism, not linked with MCPH1 locus)	Iranian
[30]	1	mis in BRCT1	nk	<−3.5 (6 m)	nk	nk	nk	prenatal cystic hygroma	nk
[31]	1	ns	−5	−4 (2 y 4 m)	normal	DD/ID (no language)	pachygyria, mild corpus callosum hypoplasia	no	Iranian (northwest)
[32]	1 (pt.1)	mis in BRCT1	−3.5	−6 (5 y 2 m)	−3	mild DD/ID	mild ventriculomegaly, small frontal lobes	mild hypotonia	Iraqi
[32]	1 (pt.2)	mis in BRCT1	−6	−5 (6 y 9 m)	−0.5 (normal)	mild DD/ID	nk	no	Turkish
[33]	1	del ex 1−11	−3	−5 (10 m)	−2	nk (infant)	coarsened gyral pattern with reduced number of sulci (normal cortex thickness and cerebellum)	small anterior fontanelle, closed posterior fontanelle	Indian
[34]	1	del ex 6−9 + 7qter del	−1.5	−5 (8 y)	−2	severe DD/ID (no language)	normal	failure to thrive, GERD, downslanted palpebral fissures, large ears, scoliosis	nk
[35]	4 related	fs	nk	−8 to −10 (10−18 y)	normal	ID (no language)	nk	aggressive behavior	Pakistani
[36]	2 sibs	splicing/del in-frame	−2.5 to −3	−5 to −12 (15−18 y)	−1 to −3	none to severe ID	normal	no	Iranian
[37]	2 related	del ex 1−2	nk	MC	nk	DD/ID	nk	nk	nk
[38]	7 related	del ex 1−2	nk	−6 to −7 (18−44 y)	normal	mild ID	nk	no	Pakistani (Punjabi)
[38]	3 related	del ex 1−11	nk	−6 to −7 (10−27 y)	normal	mild ID	nk	irritability	Pakistani (Baloch)
[39]	2 sibs	del ex 1−8	nk	−3.5 (3−14 y)	short	mild DD, ID (poor language)	normal (small lipoma)	epicanthus, esotropia, low hairline, large ears, thin upper lip (right lung hypoplasia in 1 sib)	Hispanic
[40]	2 sibs	mis central region	MC	−5 to −6 (5−10 y)	nk	severe ID (poor language)	nk	sloping forehead	Saudi
[41]	1	fs	nk	MC (1.5 m)	normal	nk (infant)	cortical atrophy, deep sulcation	long philtrum, large ears, dermatitis	Turkish
[3]	2 sibs	ns ^b^ + biallelic *TRAPPC9* mis	MC	−3 to −4 (13−16 y)	−1.5 (low normal)	DD, severe ID (poor or no language)	corpus callosum and cerebellum hypoplasia/atrophy, mild colpocephaly	hyperkinetic movements, epilepsy	Moroccan
This report	1	del ex 1−8	−3.5	−5 (10 y)	−2	mild DD, profound ID (no language)	fronto-polar simplified gyral pattern, PNH, mild ventriculomegaly, enlarged posterior fossa	sloping forehead, highly arched eyebrow, exotropia, epicanthus, large ears; hyperactivity	Pakistani (northeast)

# = number; OFC = occipital–frontal circumference; SD = standard deviations (approximated to nearest 0.5 increment); DD = developmental delay; ID = intellectual disability; MRI = magnetic resonance imaging; peds = pedigrees; sibs = siblings; nk = not known/not evaluated; ns = nonsense; fs = frameshift; mis = missense; del = deletion; ex = exon(s); y = years; m = months; MC = microcephaly; PNH = periventricular nodular heterotopia; PCC = premature chromosome condensation; GERD = gastroesophageal reflux disease. ^a^ number of affected individuals evaluated in each study (in brackets: number of independent pedigrees or individuals). ^b^ possibly generating a truncated protein missing BRCT2/3; also homozygous in a healthy sister heterozygous for the *TRAPPC9* variant: suspected digenic interaction.

## Data Availability

All data is available upon reasonable request by contacting the corresponding author.

## Supplementary Materials

The following supporting information can be downloaded at: https://www.mdpi.com/article/10.3390/genes13040634/s1, Figure S1: Genetic testing results, Figure S2: MLPA analysis of DNA samples from our proband and both parents, Table S1: Clinical findings and gene variants in MCPH1 families described in the literature.
