# Supplementary material for: MCPH1: A Novel Case Report and a Review of the Literature
Source: Genes (Basel). 2022 Apr 2;13(4):634. doi: 10.3390/genes13040634 (PMC9032034; doi:10.3390/genes13040634)

Figure S1. Genetic testing results. (**a**) Pedigree with sequence-tagged markers for the MCPH1 locus. (**b**) PCR amplification of MCPH1 exons 1-10 in proband (left) and healthy control (right). (**c**) SNP array dialog window showing a deletion of about 61kb. (**d**) Long-range PCR amplification of two con-trols, the proband and both parents with genomic primers flanking the deletion.

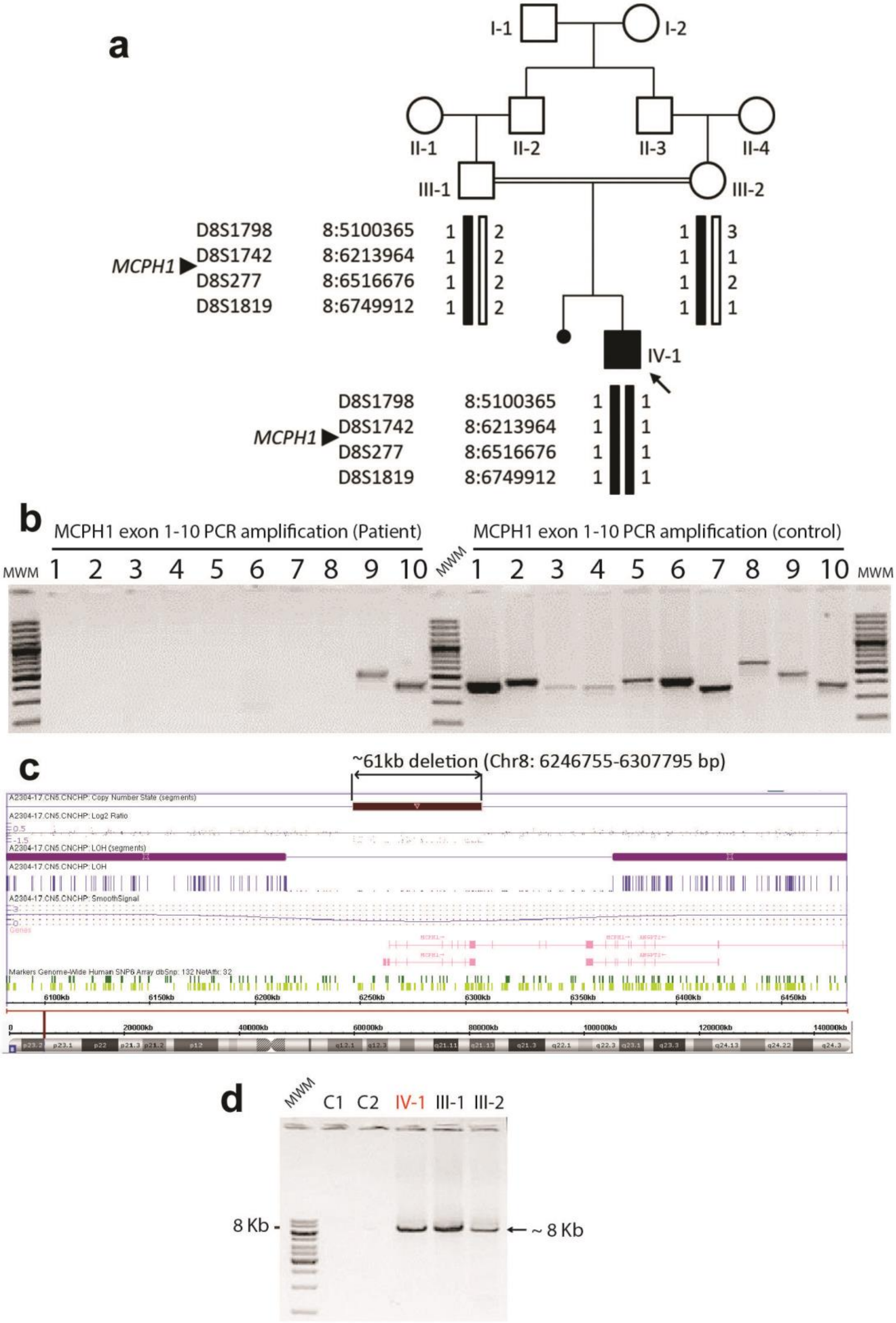

Figure S2. MLPA analysis of loci associated with primary macrocephaly in proband and parents. Ratios for probes MCPH1-2, MCPH1-4, MCPH1-8 (GRCh37 positions 6254211, 6276358, 6290246 on chromosome 8) indicate a biallelic deletion of *MCPH1* exons 1-8 in the proband and a heterozygous deletion in both parents.

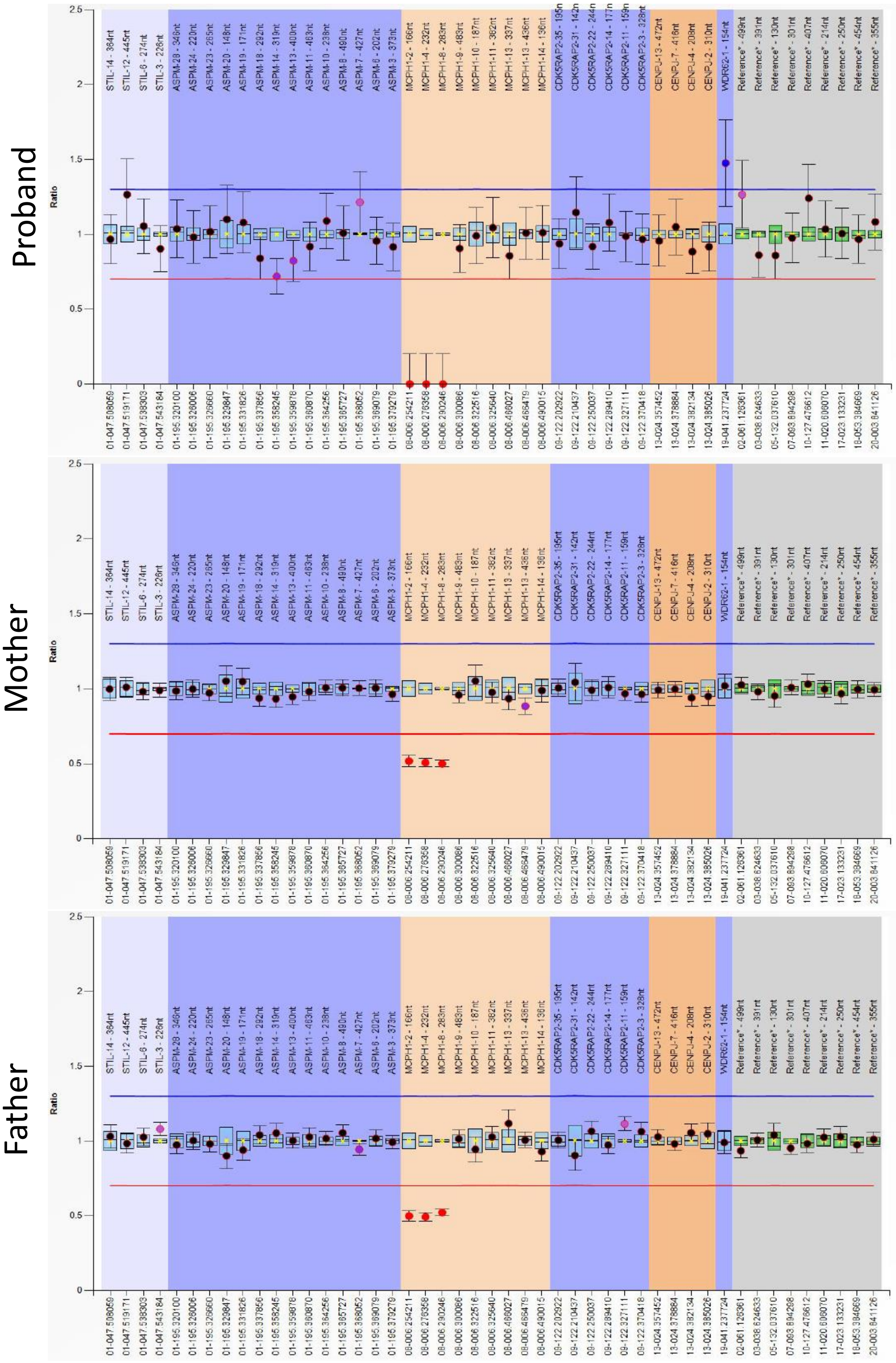

Supplement: Supplementary file 1 [file genes-13-00634-s001.zip › Figures S1-S2.pdf]
